# Assessing the Methods, Tools, and Statistical Approaches in Google Trends Research: Systematic Review

**DOI:** 10.2196/jmir.9366

**Published:** 2018-11-06

**Authors:** Amaryllis Mavragani, Gabriela Ochoa, Konstantinos P Tsagarakis

**Affiliations:** 1 Department of Computing Science and Mathematics University of Stirling Stirling, Scotland United Kingdom; 2 Department of Environmental Engineering Democritus University of Thrace Xanthi Greece

**Keywords:** big data, health assessment, infodemiology, Google Trends, medicine, review, statistical analysis

## Abstract

**Background:**

In the era of information overload, are big data analytics the answer to access and better manage available knowledge? Over the last decade, the use of Web-based data in public health issues, that is, infodemiology, has been proven useful in assessing various aspects of human behavior. Google Trends is the most popular tool to gather such information, and it has been used in several topics up to this point, with health and medicine being the most focused subject. Web-based behavior is monitored and analyzed in order to examine actual human behavior so as to predict, better assess, and even prevent health-related issues that constantly arise in everyday life.

**Objective:**

This systematic review aimed at reporting and further presenting and analyzing the methods, tools, and statistical approaches for Google Trends (infodemiology) studies in health-related topics from 2006 to 2016 to provide an overview of the usefulness of said tool and be a point of reference for future research on the subject.

**Methods:**

Following the Preferred Reporting Items for Systematic Reviews and Meta-Analyses guidelines for selecting studies, we searched for the term “Google Trends” in the Scopus and PubMed databases from 2006 to 2016, applying specific criteria for types of publications and topics. A total of 109 published papers were extracted, excluding duplicates and those that did not fall inside the topics of health and medicine or the selected article types. We then further categorized the published papers according to their methodological approach, namely, visualization, seasonality, correlations, forecasting, and modeling.

**Results:**

All the examined papers comprised, by definition, time series analysis, and all but two included data visualization. A total of 23.1% (24/104) studies used Google Trends data for examining seasonality, while 39.4% (41/104) and 32.7% (34/104) of the studies used correlations and modeling, respectively. Only 8.7% (9/104) of the studies used Google Trends data for predictions and forecasting in health-related topics; therefore, it is evident that a gap exists in forecasting using Google Trends data.

**Conclusions:**

The monitoring of online queries can provide insight into human behavior, as this field is significantly and continuously growing and will be proven more than valuable in the future for assessing behavioral changes and providing ground for research using data that could not have been accessed otherwise.

## Introduction

Big data are characterized by the 8 Vs [1]: volume (exponentially increasing volumes) [2], variety (wide range of datasets), velocity (high processing speed) [3], veracity, value [4,5], variability, volatility, and validity [1]. Big data have shown great potential in forecasting and better decision making [1]; though handling these data with conventional ways is inadequate [6], they are being continuously integrated in research [7] with novel approaches and methods.

The analysis of online search queries has been of notable popularity in the field of big data analytics in academic research [8,9]. As internet penetration is continuously increasing, the use of search traffic data, social media data, and data from other Web-based sources and tools can assist in facilitating a better understanding and analysis of Web-based behavior and behavioral changes [10].

The most popular tool for analyzing behavior using Web-based data is *Google Trends* [11]. Online search traffic data have been suggested to be a good analyzer of internet behavior, while Google Trends acts as a reliable tool in predicting changes in human behavior; subject to careful selection of the searched-for terms, Google data can accurately measure the public’s interest [12]. Google Trends provides the field of big data with new opportunities, as it has been shown to be valid [13] and has been proven valuable [14,15], accurate [16], and beneficial [17] for forecasting. Therefore, great potential arises from using Web-based queries to examine topics and issues that would have been difficult or even impossible to explore without the use of big data. The monitoring of Web-based activity is a valid indicator of public behavior, and it has been effectively used in predictions [18,19], nowcastings [20], and forecasting [17,21,22].

Google Trends shows the changes in online interest for time series in any selected term in any country or region over a selected time period, for example, a specific year, several years, 3 weeks, 4 months, 30 days, 7 days, 4 hours, 1 hour, or a specified time-frame. In addition, different terms in different regions can be compared simultaneously. Data are downloaded from the Web in “.csv” format and are adjusted as follows: “*Search results are proportionate to the time and location of a query: Each data point is divided by the total searches of the geography and time range it represents, to compare relative popularity. Otherwise places with the most search volume would always be ranked highest. The resulting numbers are then scaled on a range of 0 to 100 based on a topic’s proportion to all searches on all topics. Different regions that show the same number of searches for a term will not always have the same total search volumes* ” [23].

Healthcare is one of the fields in which big data are widely applied [24,25], with the number of publications in this field showing a high increase [26]. Researchers have placed a significant focus on examining Web-based search queries for health and medicine related topics [27]. Data from Google Trends have been shown to be valuable in predictions, detection of outbreaks, and monitoring interest, as detailed below, while such applications could be analyzed and evaluated by government officials and policy makers to deal with various health issues and disease occurrence.

The monitoring and analysis of internet data fall under the research field of infodemiology, that is, employing data collected from Web-based sources aiming at informing public health and policy [28]. These data have the advantage of being real time, thus tackling the issue of long periods of delay from gathering data to analysis and forecasting. Over the past decade, the field of infodemiology has been shown to be highly valuable in assessing health topics, retrieving web-based data from, for example, Google [29,30], Twitter [31-34], social media [35,36], or combinations of ≥2 Web-based data sources [37,38].

As the use of Google Trends in examining human behavior is relatively novel, new methods of assessing Google health data are constantly arising. Up to this point, several topics have been examined, such as epilepsy [39,40], cancer [41], thrombosis [42], silicosis [43], and various medical procedures including cancer screening examinations [44,45], bariatric surgery [46], and laser eye surgery [47].

Another trend rising is the measurement of the change in interest in controversial issues [48,49] and in drug-related subjects, such as searches in prescription [50] or illicit drugs [51,52]. In addition, Google Trends data have been used in examining interest in various aspects of the health care system [53-55].

Apart from the above, Google Trends data have also been useful in measuring the public’s reaction to various outbreaks or incidents, such as attention to the epidemic of Middle East Respiratory Syndrome [56], the Ebola outbreak [57], measles [58], and Swine flu [59], as well as the influence of media coverage on online interest [60]. Google queries for the respective terms have been reported to increase or peak when a public figure or celebrity is related [61-65].

Google Trends has also been valuable in examining seasonal trends in various diseases and health issues, such as Lyme disease [66], urinary tract infection [67], asthma [30], varicose vein treatment [68], and snoring and sleep apnea [69]. Furthermore, Deiner et al [70] showed that indeed there exists the same seasonality in Google Trends and clinical diagnoses. What has also been reported is that seasonality in Google searches on tobacco is correlated with seasonality in Google searches on lung cancer [71], while online queries for allergic rhinitis have the same seasonality as in real life cases [72]. Thus, we observe that, apart from measuring public interest, Google Trends studies show that the seasonality of online search traffic data can be related to the seasonality of actual cases of the respective diseases searched for.

As mentioned above, Google queries have been used so far to examine general interest in drugs. Taking a step further, Schuster et al [73] found a correlation between the percentage change in the global revenues in Lipitor statin for dyslipidemia treatment and Google searches, while several other studies have reported findings toward this direction, that is, correlations of Web-based searches with prescription issuing [74-76]. The detection and monitoring of flu has also been of notable popularity in health assessment. Data from Google Flu Trends have been shown to correlate with official flu data [77,78], and Google data on the relevant terms correlate with cases of influenza-like illness [79].

In addition, online search queries for suicide have been shown to be associated with actual suicide rates [80,81], while other examples indicative of the relationship between Web-based data and human behavior include the correlations between official data and internet searches in veterinary issues [82], sleep deprivation [83], sexually transmitted infections [84], Ebola-related searches [85], and allergies [86,87].

Furthermore, Zhou et al [88] showed how the early detection of tuberculosis outbreaks can be improved using Google Trends data; while suicide rates and Google data seem to be related, the former are suggested to be a good indicator for developing suicide prevention policies [89]. In addition, methamphetamine criminal behavior has been shown to be related to meth searches [90]. Finally, recent research on using Google Trends in predictions and forecasting include the development of predictive models of pertussis occurrence [91], while online search queries have been employed to forecast dementia incidence [92] and prescription volumes in ototopical antibiotics [93].

Given the diversity of subjects that Google Trends data have been used up for until this point to examine changes in interest and the usefulness of this tool in assessing human behavior, it is evident that the analysis of online search traffic data is indeed valuable in exploring and predicting behavioral changes.

In 2014, Nuti et al [27] published a systematic review of Google Trends research including the years up to 2013. This review was of importance as the first one in the field, and it reported Google Trends research up to that point. The current review differs from Nuti et al’s in two ways. First, it includes 3 more full years of Google Trends research, that is, 2014, 2015, and 2016, which account for the vast majority of the research conducted in this field for the examined period based on our selection criteria. Second, while the first part of our paper is a systematic review reporting standard information, that is, authors, country, region, keywords, and language, the second part offers a detailed analysis and categorization of the methods, approaches, and statistical tools used in each of this paper. Thus, it serves as a point of reference in Google Trends research not only by subject or topic but by analysis or method as well.

## Methods

**Figure 1:**
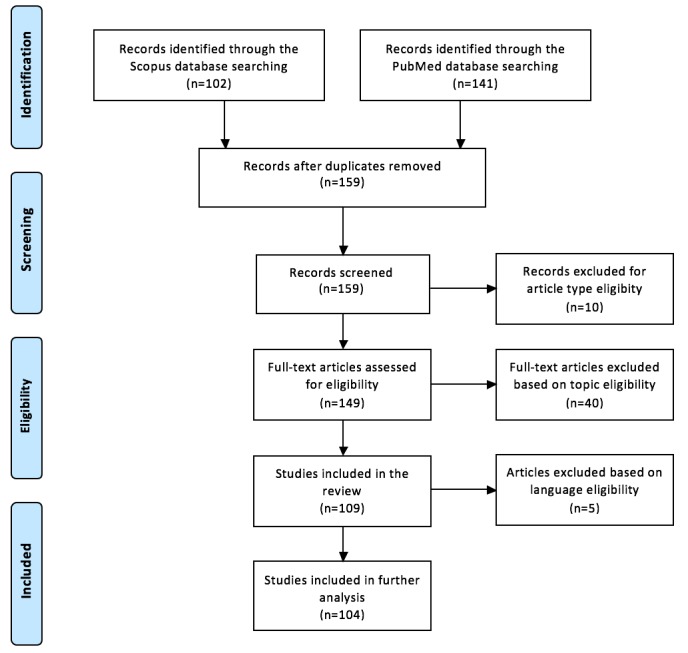
Preferred Reporting Items for Systematic Reviews and Meta-Analyses flow diagram of the selection procedure for including studies.

The aim of this review was to include all articles on the topics of health and medicine that have used Google Trends data since its establishment in 2006 through 2016. We searched for the term “Google Trends” in the Scopus [94] and PubMed [95] databases from 2006 to 2016, and following the Preferred Reporting Items for Systematic Reviews and Meta-Analyses guidelines (Figure 1), the total number of publications included in this review was 109.

**Figure 2:**
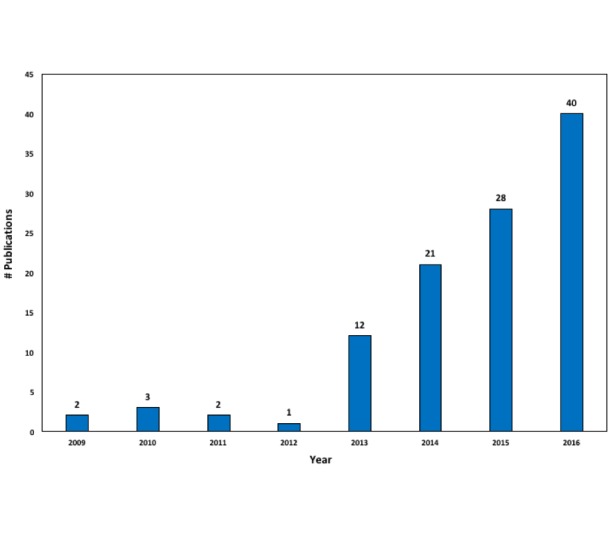
Google Trends' publications per year in health-related fields from 2009 to 2016.

First, we conducted a search in Scopus for the keyword “Google Trends” in the “Abstract-Title-Keywords” field for “Articles,” “Articles in press,” “Reviews,” and “Conference papers” from 2006 to 2016. Out of the available categories, we selected “Medicine,” “Biochemistry Genetics and Molecular Biology,” “Neuroscience,” “Immunology and Microbiology,” “Pharmacology, Toxicology, and Pharmaceuticals,” “Health Profession,” “Nursing,” and “Veterinary.” The search returned 102 publications. Second, we searched for the keyword “Google Trends” in PubMed from 2006 to 2016, which provided a total of 141 publications. Excluding the duplicates, which numbered 84 in total, 159 publications met our criteria. Excluding the ones that did not match the criteria for article type (10 publications) and the ones that did not fall inside the scope of health and medicine (40 publications), a total of 109 studies were included in this review. Note that 5 studies were written in a language other than English and were therefore not included in the quantitative part or in the detailed analysis of the methods of each study. Figure 2 depicts the number of publications by year from 2009 to 2016: 2 in 2009, 3 in 2010, 2 in 2011, 1 in 2012, 12 in 2013, 21 in 2014, 28 in 2015, and 40 in 2016.

The selected studies are further analyzed according to their methodologies, and the gaps, advantages, and limitations of the tool have been discussed so as to assist in future research. Thus, we provide a more detailed categorization of the examined papers according to the main category that they belong to, that is, visualization and general time series analysis, seasonality, correlations, predictions or forecasting, modeling, and statistical method or tool employed. Note that a study can fall into >1 category. The categorization by individual medical field is not applicable due to the high number of individual topics. Table 1 consists of the description of each parameter used to classify each study.

**Table 1:** Description of the parameters used for classification.

Parameter	Description
Authors	Includes the surname of the authors, date of publication, and link to the reference list (eg, *Smith et al, 2016 [57]*).
Period	Refers to the time-frame for which Google Trends data were retrieved and used in the study (eg, *2004-2015*).
Region	Refers to the country or countries or region (eg, *USA; Worldwide; Oceania*) that Google Trends data were extracted for.
Language	Refers to the language in which the Google Trends search was conducted (eg, search for the Italian word *Si*).
Keywords	Basic keywords are included in this category, mostly referring to the health topic examined and important keywords used to describe it.
Visualization (V)	Includes any form of visualization, that is, figures, maps, and screenshots (eg, screenshots of the Google Trends website).
Seasonality (S)	Studies that have explored the seasonality of the respective topic are included.
Correlations (C)	Studies that have examined correlations are included in this category. Correlations may be between Google Trends data and official data, among Google Trends time series, or between Google Trends and other Web-based sources’ time series.
Forecasting (F)	This category includes studies that conducted forecasting of either Google Trends time series or diseases, outbreaks, etc, using Google Trends data, independent of the method used.
Modeling (M)	Studies in this category conducted some form of modeling using Google Trends data.
Statistical Tools (St)	This category includes the studies that used statistical tools or tests, eg, *t* test. Tools and methods for statistical modeling, (eg, *regression*), are not included in this category but only in the category of Modeling.

## Results

Multimedia Appendix 1 consists of the first classification of the selected studies [27,39-57,59-93,96-144]; there are 104 in total, as the studies of Kohler et al [145], Orellano et al [146], Cjuno et al [147], Tejada-Llacsa [148], and Yang et al [149] are written in German, Spanish, or Chinese, and thus are not included in the more detailed categorization and analysis.

All the examined papers involve, by definition, time series analysis, and almost all include some form of visualization. Only 8.7% (9/104) studies used Google Trends data for predictions and forecasting, and 23.1% (24/104) used them for examining seasonality, while correlations and modeling were performed in 39.4% (41/104) and 32.7% (34/104) studies, respectively. As the category of forecasting and predictions exhibits the least number of studies, it is evident that a gap exists in the literature for forecasting using Google Trends in health assessment.

**Figure 3:**
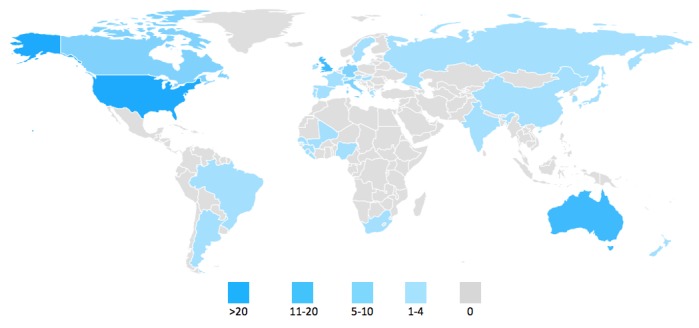
Countries by number of Scopus and PubMed publications using Google Trends.

As is evident in Multimedia Appendix 1, Google queries have been employed up to this point in many countries and several languages. Figure 3 shows a worldwide map by examined country for assessing health and medicine related issues using Google Trends data up to 2016. Worldwide, the studies that explore topics related to the respective terms number 23 in total. As far as individual countries are concerned, US data have been employed in the most (60) studies, while other countries that have been significantly examined include the United Kingdom (15), Australia (13), Canada (9), Germany (8), and Italy (7).

The four most examined countries are English-speaking ones. The reasons for this could include that Google Trends, though not case-sensitive, does take into account accents and spelling mistakes; therefore, for countries with more complicated alphabets, the analysis of Web-based data should be more careful. In addition, other factors that could play a significant role and are taken into account when choosing the countries to be examined using online search traffic data are the availability of official data, the openness of said data, any internet restrictions or monitoring in countries with lower scores in freedom of press or freedom of speech, and internet penetration.

The rest of the analysis consists of the further breaking down of the initial categorization to include the respective methods that were used for examining seasonality, correlations, forecasting, and performing statistical tests and estimating models, along with a concise introduction to each of these methods and how they were used to assess health issues.

Table 2 shows the methods used to explore seasonality; Tables 3 and 4 present the methods used to examine correlations and perform predictions and forecasting, respectively. Finally, Tables 5 and 6 list the modeling methods and other statistical tools employed in health assessment using Google Trends.

The most popular way to explore seasonality is to use visual evidence and examine and discuss peaks, as shown in Table 2. Furthermore, several studies have used cosinor analysis [8,69,134,138,142], which is a time series analysis method for seasonal data using least squares.

Apart from seasonality [122], analysis of variance (ANOVA) has been also used for geographical comparisons between regions or countries [49,51,68,93] and between differences in monthly data [41]. It is a test used for examining if significant differences between means exist. In the case of 2 means, *t* test is the equivalent to ANOVA.

The Kruskal-Wallis test is also a popular method for examining seasonality using Google Trends [57,68,113]. It is a nonparametric, independent of distribution test, for continuous as well as ordinal-level dependent variables, employed when the one-way ANOVA assumptions do not hold, that is, for examining statistically significant differences between ≥3 groups. It uses random sample with independent observations, with the dependent variable being at least ordinal.

Other methods of exploring seasonality include the nonparametric tests (independent of distribution) Wilcoxon signed rank [18,113] and Mann-Whitney U test [67], which are used for comparing data in different seasons or time periods when the equivalent parametric *t* tests cannot be used. The latter has been also used by some studies to compare weekly data [105] and differences among regions [113].

For examining correlations (Table 3), the vast majority of the studies used the Pearson correlation coefficient, which examines the strength of association between 2 quantitative, continuous variables, employed when the relationship is linear. The Spearman rho (rank-order) correlation, the second most used method, is the nonparametric version of the Pearson correlation, has also been used to explore seasonality between time series [70]. Spearman correlation coefficient (denoted by *ρ* or *r*_*s*_) measures the levels to which 2 ranked variables (ordinal, interval, or ratio) are related to each other.

Cross-correlations are used for examining the relationship of 2 time series, while simultaneously exploring if the data are periodic. It is often employed in correlating Google Trends data with observed data [50,82,90,135] and between different Google search terms [80], while it can be also used for examining linear and temporal associations of seasonal data [71]. Cross-correlations have been also used in forecasting, where Wang et al [92] showed that cross-correlations of new dementia cases with Google Trends data can assist with the forecasting of dementia cases, and Solano et al [80] forecasted the suicide rates 2 years ahead using Google queries. The autocorrelations are basically cross-correlations for one time series, that is, a time series cross-correlated with itself.

The Kendall’s tau-b test correlation coefficient is a nonparametric alternative to Pearson and Spearman correlations and is used to measure the strength and direction of the relationship between 2 (at least ordinal) variables. It has been employed by 1 study [138] to examine the correlations between Google Trends data and the results of a paper interview survey.

The Spearman-Brown prediction (or prophecy) formula is used to predict how reliable the test is after changing its length. It has also been employed by only 1 study [65] to explore the relationship between railway suicide and Google hits.

The generalized linear model estimates the linear relationship between a dependent and ≥1 independent variables. It was used by Domnich et al [79] to predict influenza-like illness morbidity, with the exploratory variables being “Influenza,” “Fever,” and “Tachipirin search volumes,” along with the Holt-Winters method and the autoregressive moving average process for the residuals. Holt-Winters is a method employed in exploring the seasonality in time series, and for predictions, the autoregressive moving average (also called the Box-Jenkins model) is a special case of the autoregressive integrated moving average, used for the analysis of time series and predictions.

Autoregressive integrated moving average is a commonly used method for time series analysis and predictions [55,63,86,92,141], the latter having also been assessed by linear regressions and modeling [88,91]. Multivariable regressions are used to estimate the relationship of ≥2 independent variables with a dependent one. In Google Trends, they have been used to relate Ebola searches, reported cases, and the Human Development Index [85] and to study the relationship between climate and environmental variables and Google hits [125].

Hierarchical linear modeling is a regression of ordinary least squares that is employed to analyze hierarchically structured data, that is, units that are grouped together, and it has been employed by 1 study so far [83].

The Mann-Kendall test, which is the nonparametric alternative test to the independent sample, has been used to show the statistical differences of peaks [43] and to detect trends [140]. Finally, the *t* test is used to compare 2 sample means of the same population, and it has been employed for comparing Google searches with the baseline period [105] and to examine the statistical differences of peaks [41].

**Table 2:** Methods for exploring seasonality with Google Trends in health assessment.

Number	Authors	Method	Description
1	Bakker et al, 2016 [96]	Morlet Wavelet Analysis	To test the seasonality of Google Trends data in the examined countries
2	Braun and Harreus, 2013 [104]	Visual evidence	N/A^a^
3	Crowson et al, 2016 [93]	Seasonal peaks	N/A
4	Deiner et al, 2016 [70]	Spearman correlation	Correlating the seasonality of clinical diagnoses with Google Trends data
5	El-Sheikha, 2015 [113]	Kruskal-Wallis test	To show seasonality for different months
6	Garrison et al, 2015 [116]	Least-squares sinusoidal model	Variability in outcomes (supported also from a comparison with searches in Australia)
7	Harsha et al, 2014 [68]	Kruskal-Wallis test	Seasonal (monthly) comparisons
8	Harsha et al, 2015 [119]	Kruskal-Wallis test	Seasonal (monthly) comparisons
9	Hassid et al, 2016 [120]	Pearson correlation	To examine seasonal variations across symptoms
10	Ingram and Plante, 2013 [122]	Cosinor analysis; analysis of variance	To test the seasonal variation of the normalized Google Trends data; to compare the seasonal increase among the examined countries
11	Ingram et al, 2015 [69]	Cosinor analysis	To test the seasonal variation of the normalized Google Trends data
12	Kang et al, 2015 [72]	Visual observation	N/A
13	Leffler et al, 2010 [125]	Correlations	Showing correlations among the 4 seasons for the 39 examined terms
14	Liu et al, 2016 [127]	Seasonal model and a null model	Seasonality explained the searches significantly better with an F-test
15	Phelan et al, 2016 [133]	Correlograms (autocorrelations plots)	Visual interpretation for exploring seasonal peaks
16	Plante and Ingram, 2014 [134]	Cosinor analysis	To test the seasonal variation of the normalized Google Trends data
17	Rossignol et al, 2013 [67]	Mann-Whitney U test; Harmonic Product Spectrum	Comparison of summer vs winter hits; evaluation of seasonality
18	Seifter et al, 2010 [66]	Visual evidence	N/A
19	Sentana-Lledo et al, 2016 [138]	Cosinor analysis	To test the seasonal variations of the Google Trends data
20	Takada, 2012 [139]	Visual evidence	N/A
21	Telfer and Woodburn, 2015 [140]	Two-way Wilcoxon signed rank test	To explore differences between winter and summer
22	Toosi and Kalia, 2015 [142]	Visual evidence; cosinor analysis	To identify differences in seasonality between countries
23	Willson et al, 2015 [86]	Visual evidence	N/A
24	Zhang et al, 2015 [71]	Periodograms; ideal pass filter	To study the periodograms; to extract seasonal components

^a^N/A: not applicable.

Many studies have employed Google Trends for visualizing the changes in online interest or discussing peaks and spikes [60,62,123,124]. Brigo and Trinka [40] and Brigo et al [39] have studied the search volumes for related terms, Chaves et al [109] and Luckett et al [128] have explored terms related to the studied topic, and Davis et al [110] have examined related internet searches. Other approaches include the reporting of the polynomial trend lines [46] and investigation of statistically significant differences in yearly increases [119]. In addition, “Google Correlate” has been used to explore related terms [91,138].

Finally, several studies have used other sources of big data, namely, Google News [43,63,80], Twitter [43,54,61,63,108], Yandex [52], Baidu [121], Wikipedia [43,63], Facebook and Google+ [54], and YouTube [43,54,63]. Google is the most popular search engine. However, other Web-based sources are used or even preferred to Google in some regions; therefore, many studies use data from these sources to examine general interest in the respective subjects, compare them to Google Trends data, or use them together as variables.

**Table 3:** Methods of exploring correlations using Google Trends in health assessment.

Number	Authors	Method	Description
1	Alicino et al, 2015 [85]	Pearson correlation	Ebola-related Google Trends data with Ebola cases
2	Arora et al, 2016 [81]	Spearman correlation	Suicide search activity vs official suicide rates (and per age)
3	Bakker et al, 2016 [96]	Correlations	Between Google Trends data and reported cases
4	Bragazzi et al, 2016 [99]	Pearson correlation	Between Google Trends data and epidemiological data
5	Bragazzi, 2013 [98]	Autocorrelation; Pearson correlation	For the time series for multiple sclerosis (MS); between MS terms
6	Bragazzi et al, 2016 [101]	Autocorrelation; Partial Autocorrelation	To compute correlation of the time series with its own values
7	Bragazzi et al, 2016 [102]	Pearson correlation	Status epilepticus terms with etiology and management related terms
8	Bragazzi et al, 2016 [43]	Pearson correlation	Google searches for Silicosis with Normalized Google News, Google Scholar, PubMed Publications, Twitter traffic, Wikipedia
9	Bragazzi et al, 2016 [63]	Pearson correlation	Among Google Trends data and other data generating sources
10	Bragazzi, 2014 [103]	Pearson correlation; autocorrelation and partial autocorrelation	Nonsuicidal self-injury and related terms; nonsuicidal self-injury plots showed regular cyclical pattern
11	Cavazos-Regh et al, 2015 [107]	Pearson correlation	Among Google Trends data for noncigarette tobacco and prevalence
12	Cho et al, 2013 [78]	Pearson correlation	Google flu-related queries with surveillance data for different influenza seasons
13	Crowson et al, 2016 [93]	Pearson correlation	Between the selected keywords. Between medical prescriptions data and Google Trends data
14	Deiner et al, 2016 [70]	Spearman correlation	For correlating seasonality of clinical diagnoses with Google Trends data
15	Domnich et al, 2015 [79]	Pearson correlation	Among the examined search terms and influenza-like illness
16	Foroughi et al, 2016 [115]	Rank correlations; cross-country correlations; Pearson correlations	For search volumes; for the search volumes for cancer; for the weekly search volumes between countries
17	Gahr et al, 2015 [75]	Pearson correlation	Among annual prescription volumes and Google Trends data
18	Gamma et al, 2016 [90]	Cross-correlations	Cross-correlations between search volumes and crime statistics
19	Gollust et al, 2016 [117]	Multinomial Logit Models	To relate health insurance rates
20	Guernier et al, 2016 [82]	Spearman correlation; cross-correlation	Correlating the examined search terms with notifications of tick paralysis cases record; with lag values from −7 to +7 months
21	Hassid et al, 2016 [120]	Pearson correlation	Between Google Trends data and National Inpatient Sample data
22	Johnson et al, 2014 [84]	Pearson correlation	Pearson correlations to explore the relation of Google Trends data and sexually transmitted infection reported rates
23	Kang et al, 2013 [77]	Pearson correlation	To explore the association of (and among) search terms with surveillance data
24	Kang et al, 2015 [72]	Spearman correlation	Google Trends data for allergic rhinitis and related Google Trends terms and real world epidemiologic data for the United States
25	Koburger et al, 2015 [65]	Spearman-Brown correlation	To explore relations among Google Trends data and railway suicides
26	Ling and Lee, 2016 [126]	Pearson correlation	Between disease prevalence and Google Trends data
27	Mavragani et al, 2016 [76]	Pearson correlation	Between Google Trends data and published papers and Google Trends data with prescriptions
28	Phelan et al, 2016 [133]	Linear Regression	To examine if there is significant correlation between searches and time
29	Poletto et al, 2016 [56]	Pearson correlation	Between Google Trends data and number of alerts published by ProMED mail and the number of Disease Outbreak News published by the World Health Organization
30	Pollett et al, 2015 [91]	Pearson correlation	To shortlist related search terms to pertussis
31	Rohart et al, 2016 [135]	Spearman rank correlations; Spearman correlation; cross-correlations	For the diseases examined; correlations between diseases and the investigated search metrics; to identify best lags
32	Shin et al, 2016 [137]	Spearman correlation	Between Google Trends data and the number of confirmed cases of Middle East Respiratory Syndrome and for quarantined cases of Middle East Respiratory Syndrome
33	Schootman et al, 2015 [45]	Pearson correlation	Between Respiratory Syncytial Virus and Behavioral Risk Factor Surveillance System prevalence data for 5 cancer screening tests
34	Schuster et al, 2010 [73]	Correlations	Lipitor Google Trends data and Lipitor revenues
35	Sentana-Lledo et al, 2016 [138]	Kendall’s Tau-b test	To explore the correlation of Google Trends data with paper interview survey results
36	Simmering et al, 2014 [50]	Cross-correlations	Between Google Trends data for drugs and drug utilization, to see changes in search volumes following knowledge events
37	Solano et al, 2016 [80]	Correlations; cross-correlations	Between Google Trends data for suicide and national suicide rates; between different search terms
38	Wang et al, 2015 [92]	Pearson correlation	Between Google Trends data and new dementia cases
39	Willson et al, 2015 [86]	Spearman correlation	Between Google Trends data and observed data for aeroallergens
40	Zhang et al, 2015 [71]	Cross-correlations	To examine linear and temporal associations of the seasonal data
41	Zhang et al, 2016 [51]	Pearson correlation	To study pairwise comparisons among searches for different terms in Google Trends

**Table 4:** Forecasting and predictions using Google Trends in health assessment.

Number	Authors	Method	Description
1	Bakker et al, 2016 [96]	Statistical model	For forecasting chicken poxforce of infection, that is, monthly per capita rate of infection of children 0-14
2	Domnich et al, 2015 [79]	Generalized least squares (maximum likelihood estimates); Holt-Winters	Query-based models to predict influenza-like illness morbidity, with the exploratory variables: Influenza, Fever, Tachipirin; compared for forecasting power with Holt-Winters based on the real data (hold out set)
3	Parker et al, 2016 [132]	Statistical model	For forecasting deaths for 1 year in advance (2015)
4	Pollett et al, 2015 [91]	Prediction model	Tested the predicted model with a left-out dataset for prediction accuracy
5	Rohart et al, 2016 [135]	Linear models	To forecast with 1 or 2 weeks step
6	Solano et al, 2016 [80]	Cross-Correlations	Forecasting for suicides for 2 years without data (2013-14) based on Google Trends data of those years
7	Wang et al, 2015 [92]	Cross-Correlations	To investigate forecasting with lags of 0-12 months
8	Zhang et al, 2016 [51]	Autoregressive Moving Average	To predict Respiratory Syncytial Virus for “dabbing”
9	Zhou et al, 2011 [88]	Dynamic model	To provide real time estimations by correcting the forecasting with the new morbidity data when published

**Table 5:** Statistical modeling using Google Trends in health assessment.

Number	Authors	Method	Description
1	Alicino et al, 2015 [85]	Multivariate regression	For relating Ebola Google Trends data, number of Ebola Cases, and the Human Development Index
2	Bakker et al, 2016 [96]	Statistical model	For forecasting chicken poxforce of infection, that is, monthly per capita rate of infection
3	Bentley and Ormerod, 2009 [59]	Maximum likelihood estimation	Established social model for engaging a new behavior for Web-based searching for flu terms
	Barnes et al, 2015 [83]	Hierarchical linear modeling	Three levels: 3 Mondays, 6 years, 47 search terms
4	Bragazzi, 2013 [98]	Multiple linear regression	To confirm multiannual long-term trends
5	Domnich et al, 2015 [79]	Generalized linear model, autoregressive moving average process	Query volume-based models to predict influenza-like illness morbidity
6	El-Sheikha, 2015 [113]	Linear regression	To show the global, regional, and country level interest for the search term
7	Fenichel et al, 2013 [114]	Moving average, generalized linear model	Google Trends data as a variable in predicting loses in flights
8	Garrison et al, 2015 [116]	Seasonal model	Best fit combination of a straight line and a sinusoid
9	Gollust et al, 2016 [117]	Multinomial logit models	To relate health insurance rates
10	Haney et al, 2014 [55]	ARIMA^a^	Radiology residency interest
11	Harsha et al, 2014 [68]	Linear model	Statistical justification of annual increase in search volumes
12	Harsha et al, 2015 [119]	Linear model	Statistical justification of annual increase in search volumes and of the Web-based interest related to applications for interventional radiology
13	Leffler et al, 2010 [125]	Multivariable Linear Regressions	For studying the effect of climatic and environmental variables to internet searches
17	Linkov et al, 2014 [46]	Polynomial trend lines	Fitted spline polynomial trend lines per time without statistical reporting
18	Liu et al, 2016 [127]	Seasonal model	Best fit combination of a straight line and a sinusoid
19	Majumder et al, 2016 [129]	Linear Smoothing	To adjust HealthMap to using Google Trends, model fits
20	Noar et al, 2013 [64]	Linear Regression	To estimate the slope coefficient for changes in the magnitude of the effect size of Google Trends data and media search increases
21	Parker et al, 2016[132]	L1-regularization on Google Trends	To build a model for forecasting deaths in each state
22	Phelan et al, 2014 [49]	Linear Regression	To estimate the relation between news reports and search activity
23	Phelan et al, 2016 [133]	Linear Regression	To examine if there is a significant correlation between searches and time
24	Pollett et al, 2015 [91]	Linear Regression	Prediction model for pertussis cases based on Google Trends data of the most related terms
25	Rohart et al, 2016 [135]	Linear models	To forecast with 1 or 2 weeks step
26	Scatà et al, 2016 [136]	Epidemic model	Google Trends data is a measure of awareness, along with other sources
27	Schuster et al, 2010 [73]	Generalized Linear models	Google Trends data for the examined drugs, Google Trends data and changes in annual revenues, and Google Trends data vs resource utilization
28	Stein et al, 2013 [47]	Regression Fit Lines	To examine differences in queries
29	Telfer and Woodburn, 2015 [140]	Visual decomposition; local regression	Figures 4, 6 and 8; regression-based decomposition of the time series for the search terms
30	Troelstra et al, 2016 [141]	ARIMA	To account for dependency between data points in time series for “quit smoking” searches
31	Willson et al, 2015 [86]	ARIMA	To quantify the effect of the observed (pollen) counts with the levels of search activity
32	Willson et al, 2015 [87]	ARIMA	To quantify the effect of the observed (pollen) counts with the levels of search activity
33	Yang et al, 2015 [144]	Prediction model (ARGO^b^)	To predict influenza-like illness
34	Zhou et al, 2011 [88]	Dynamic Modeling	For forecasting tuberculosis incidents using Google Trends data

^a^ARIMA: autoregressive integrated moving average.

^b^ARGO: autoregression with Google search data.

**Table 6:** Statistical tests and tools using Google Trends in health assessment.

Number	Authors	Method	Description
1	Bragazzi et al, 2016 [43]	Mann-Kendall test	To show the statistical difference of peaks from the remaining period
2	Bragazzi et al, 2016 [63]	ARIMA^a^	To show increased web searches due to an event, and correct seasonality
3	Campen et al, 2014 [105]	Independent samples *t* test; Mann-Whitney U test with Bonferroni correction	For comparing searches with baseline period; for multiple weekly data comparisons
4	Crowson et al, 2016 [93]	ANOVA^b^ (Post-hoc Tukey test)	To compare grouped geographical federal regions of the United States (Northeast, Midwest, South, West)
5	El-Sheikha, 2015 [113]	Wilcoxon rank test; Mann-Whitney	To study the change of interest at different time periods; to compare Web-based interest between the Northern and Southern hemispheres
6	Gahr et al, 2015 [75]	Coefficients of determination	To determine the amount of variability between annual prescription volumes and Google search terms
7	Harsha et al, 2014 [68]	ANOVA (Tukey-Kramer post hot test)	For the comparisons of US regions
8	Murray et al, 2016 [41]	ANOVA; *t* test	To explore differences in months’ means per year; for the statistical differences of peaks compared with the remaining hits
9	Noar et al, 2013 [64]	Augmented Dickey-Fuller tests	To test for nonstationarity of the time series
10	Phelan et al, 2014 [49]	ANOVA	To explore differences among countries
11	Rohart et al, 2016 [135]	Mean Square Error for Prediction	To assess prediction accuracy
12	Telfer and Woodburn, 2015 [140]	Mann-Kendall trend tests	To detect trends significantly larger than the variance in the data for search terms
13	Troelstra et al, 2016 [141]	ARIMA	Studied the effect of smoking cessation policies with ARIMA interrupted time series modeling (Multimedia Appendix 1)
14	Zhang et al, 2015 [71]	Augmented Dickey-Fuller test	To detect whether or not the extracted seasonal components of the studied trends were stationary
15	Zhang et al, 2016 [51]	ANOVA	To examine the search interest for dabbing between groups of legal status states in the United States

^a^ARIMA: autoregressive integrated moving average.

^b^ANOVA: analysis of variance.

## Discussion

### Principal Findings

With internet penetration constantly growing, users’ Web-based search patterns can provide a great opportunity to examine and further predict human behavior. In addressing the challenge of big data analytics, Google Trends has been a popular tool in research over the past decade, with its main advantage being that it uses the revealed and not the stated data. Health and medicine are the most popular fields where Google Trends data have been employed so far to examine and predict human behavior. This review provides a detailed overview and classification of the examined studies (109 in total from 2006 through 2016), which are then further categorized and analyzed by approach, method, and statistical tools employed for data analysis.

**Figure 4:**
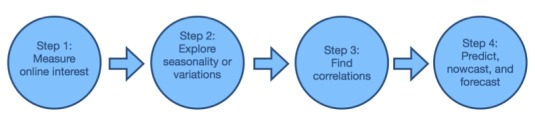
The four steps toward employing Google Trends for health assessment.

The vast majority of studies using Google Trends in health assessment so far have included data visualization, that is, figures, maps, or screenshots. As discussed in the analysis, the most popular way of using Google Trends data in this field is correlating them with official data on disease occurrence, spreading, and outbreaks. The assessment of suicide tendencies and (prescription or illegal) drug-related queries has been of notably growing popularity over the course of the last years. As is evident, the gap in the existing literature is the use of Google Trends for predictions and forecasting in health-related topics and issues. Though data on reported cases of various health issues and the respective Google Trends data have been correlated in a large number of studies, only a few have proceeded with forecasting incidents and occurrences using online search traffic data.

In research using Google Trends in health and medicine from 2006 to 2016, the ultimate goal is to be able to use and analyze Web-based data to predict and provide insight to better assess health issues and topics. The four main steps, based on the presentation of the papers published up to this point in assessing health using Google Trends, are as follows (Figure 4):

1. Measure the general Web-based interest.
2. Detect any variations or seasonality of Web-based interest, and proceed with examining any relations between actual events or cases.
3. Correlate Web-based search queries among them or with official or actual data and events.
4. Predict, nowcast, and forecast health-related events, outbreaks, etc.

### Limitations

This review followed the Preferred Reporting Items for Systematic Reviews and Meta-Analyses guidelines for selecting the examined papers from the Scopus and PubMed databases. Though this includes the majority of papers published on the topic from 2006 to 2016, the studies that are not indexed in these databases or are not indexed based on the selection criteria used in this review were not included in further analysis. In addition, as is evident in Figure 2, research using Google Trends data has shown a significant increase from each year to the next since 2013. This review included studies published in Google Trends research through 2016. However, there are several studies published in 2017 and 2018 that are not included. This review provides, at first, an overall description of each examined study, which is standard review information. The second part is a classification and assessment of the methodology, tools, and results of each study. Though the first part mainly reports what is included in the methodology of each study, the second part could include a bias, as it is the authors’ assessment and categorization of the methods employed based on the results obtained after a very careful and thorough examination of each individual study.

### Conclusions

This review consists of the studies published from 2006 to 2016 on Google Trends research in the Scopus and PubMed databases based on the selected criteria. The aim of this review was to serve as a point of reference for future research in health assessment using Google Trends, as each study, apart from the basic information, for example, period, region, language, is also categorized by the method, approach, and statistical tools employed for the analysis of the data retrieved from Google Trends. Google Trends data are being all the more integrated in infodemiology research, and Web-based data have been shown to empirically correlate with official health data in many topics. It is thus evident that this field will become increasingly popular in the future in health assessment, as the gathering of real time data is crucial in monitoring and analyzing seasonal diseases as well as epidemics and outbreaks.

## Appendix

Multimedia Appendix 1 Publication details and categorization.

## Abbreviations

ANOVA: analysis of variance
ARIMA: autoregressive integrated moving average.
MS: multiple sclerosis
