# Supplementary material for: Assessing the Methods, Tools, and Statistical Approaches in Google Trends Research: Systematic Review
Source: J Med Internet Res. 2018 Nov 6;20(11):e270. doi: 10.2196/jmir.9366 (PMC6246971; doi:10.2196/jmir.9366)
Supplement: Multimedia Appendix 1 [file jmir_v20i11e270_app1.pdf]

Multimedia Appendix 1. Publication details and categorization.

	Authors	Period	Region	Keywords	V	S	C	F	M	St	Languages
1	Alicino et al 2015	2013-2015	Guinea, Sierra Leone, Liberia, Nigeria, Mali, Senegal, USA, Spain, UK, Italy	Ebola	✔		✔		✔		English, African languages (no data)
2	Arora et al 2016	2004-2013	UK	Suicide	✔		✔				English
3	Bakker et al 2016	2004-2015	Worldwide (36 Countries)	Chicken Pox, Varicella Zoster Virus, Vaccination	✔	✔	✔	✔	✔		Multiple Languages
4	Barnes et al 2015	2008-2013	USA	Sleep, Moral Awareness	✔				✔		English
5	Bentley & Ormerod, 2009	2005, 2009	Worldwide	Bird Flu, Swine Flu	✔				✔		English
6	Borron et al 2016	2009-2015	USA	Loperamide	✔						English
7	Bragazzi, 2013	2004-2012	Italy	Multiple Sclerosis	✔		✔		✔		Italian
8	Bragazzi et al 2016	2004-2010	USA	Silicosis	✔		✔				English
9	Bragazzi et al 2016	2004-2015	Worldwide	Vaccination	✔						English
10	Bragazzi et al 2016	2004-2015	Italy	West Nile Virus	✔		✔				Italian
11	Bragazzi et al 2016	2004-2015	Worldwide	Epilepsy	✔		✔				English
12	Bragazzi et al 2016	2004-2015	Worldwide	Silicosis	✔		✔			✔	English
13	Bragazzi et al 2016	2004-2016	Worldwide, USA	Vasculitis, Autoimmune Diseases, Celebrity	✔		✔			✔	English
14	Bragazzi, 2014	2004-2012	Italy	Non-Suicidal Self Injury (NSSI)	✔		✔				Italian
15	Braun & Harreus, 2013	2005-2012	Germany	Otolaryngology, Sinusitis	✔	✔					German
16	Brigo & Trinka, 2015	2004-2014	Worldwide	Epilepsy	✔						English
17	Brigo et al 2014	2004-2013	Worldwide	Epilepsy	✔						English
18	Brigo et al 2014	2004-2013	Worldwide	Multiple Sclerosis, Epilepsy, Dementia	✔						English
19	Van Campen et al 2014	2004-2013	Netherlands, USA, UK	Epilepsy, Seizures	✔					✔	English, Dutch

	Authors	Period	Region	Keywords	V	S	C	F	M	St	Languages
20	Carneiro & Mylonakis, 2009	2004-2009	Worldwide, USA	Influenza, Flu, West Nile virus, Bird Flu, Respiratory Syncytial Virus (RSV), Bird Flu	✔						English
21	Cavazos-Regh et al 2015	2011	USA	Tobacco	✔		✔				English
22	Cha & Stow, 2015	2004-2014	USA	Toledo Water Crisis, Algae	✔						English
23	Chaves et al 2015	2004-2012	Worldwide	Tele-health, Newborn Hearing Screening							Portuguese
24	Cho et al 2013	2007-2012	South Korea	Influenza (Flu, New Flu, Swine Flu, New Influenza, Fever, Tamiflu)	✔		✔				Korean
25	Crowson et al 2016	2008-2015	USA	Otitis Externa, Ototopicals, Ciprodex, Cortisporin, Ofloxacin	✔	✔	✔			✔	English
26	Davis et al 2015	2005-2015	Worldwide	Interstitial Cystitis, Painful Bladder Syndrome	✔						English
27	Fazeli et al 2014	2004-2014	USA	Breast Cancer, Dense Breast	✔						English
28	Deiner et al 2016	2012-2014	USA, Australia	Pink Eye, Eye Allergy, Flu, Eye Drops, Eye Diseases	✔	✔	✔				English
29	DeVilbiss & Lee, 2014	2004-2014	USA	Autism Awareness, Autism, Asperger's, ADHD	✔						English
30	Domnich et al 2015	2011-2015	Italy	Influenza, Fever, Cough, Tachipirina, Paracetamol	✔		✔	✔	✔		Italian
31	El-Sheikha, 2015	2006-2013	Worldwide, 44 countries	Varicose Vein Syndrome	✔	✔			✔	✔	English, 32 languages
32	Fenichel et al 2013	2008-2010	USA	Pandemic Influenza, Swine Flu	✔				✔		English
33	Fond et al 2015	2005-2014	Worldwide	Suicide, Depression, Bipolar	✔						English
34	Foroughi et al 2016	2004-2015	Australia, Canada, New Zealand, UK, USA	Cancer	✔		✔				English
35	Gafson & Giovannoni, 2014	2008-2012	Worldwide	Chronic Cerebrospinal Venous Insufficiency	✔						English

	Authors	Period	Region	Keywords	V	S	C	F	M	St	Languages
36	Gahr et al 2015	2004-2013	Germany	Antidepressants, Prescriptions	✔		✔		✔		German
37	Gamma et al 2016	2004-2016	Switzerland, Germany, Austria	Drugs, Methamphetamine Crime	✔		✔				German (same in English)
38	Garrison et al 2015	2004-2012	USA, Australia	Leg cramps	✔	✔			✔		English
39	Gollust et al 2016	2013-2014	USA	Affordable Care Act, Health Insurance, Obamacare	✔		✔		✔		English
40	Guernier et al 2016	2011-2013	Australia	Veterinary Diseases, Tick Paralysis	✔		✔				English
41	Haney et al 2014	2004-2013	USA	Radiology Residency, Radiology Salary	✔				✔		English
42	Harorli & Harorli, 2014	2004-2014	Worldwide	Oral problems	✔						English
43	Harsha et al 2014	2004-2012	USA	Varicose Vein Syndrome	✔	✔			✔	✔	English
44	Harsha et al 2015	2006-2013	USA	Interventional Radiology, Fellowships	✔	✔			✔		English
45	Hassid et al 2016	2008-2011	USA	Gastrointestinal Symptoms	✔	✔	✔				English
46	Hossain et al 2016	2014	Guinea, Liberia, Sierra Leone, USA, UK	Ebola, Flu	✔						English
47	Huang et al 2013	2009-2011	China	Smoking, Smoking Ban, Electronic Cigarette	✔						Chinese
48	Huesch et al 2014	2012-2013	USA	Public Hospitals, Quality, Ratings	✔						English
49	Ingram & Plante, 2013	2004-2012	USA, Australia, UK, Canada, Germany	Restless Legs Syndrome	✔	✔					English
50	Ingram et al 2015	2006-2012	USA, Australia	Breathing Sleep Disorder	✔	✔					English
51	Jha et al 2015	2004-2015	USA	Oral Bisphosphonate, Prescriptions, Hip Fractures, Fosamax	✔						English
52	Johnson et al 2014	2005-2011	USA	Sexually Transmitted Infections	✔		✔				English
53	Kadry et al 2011	2010	USA	Physician Rating							English
54	Kang et al 2013	2008-2011	China	Influenza, ILI, Flu, H1N1	✔		✔				Chinese

	Authors	Period	Region	Keywords	V	S	C	F	M	St	Languages
55	Kang et al 2015	2008-2013	USA, UK, Australia	Allergic Rhinitis, Allergic Rhinitis, Pollen count, Claritin, Zyrtec	✔	✔	✔				English
56	Koburger et al 2015	2009-2010	Germany, Austria, Hungary, Netherlands, Slovenia	Suicide, Robert Enke	✔		✔				German, Hungarian, Dutch, Slovenian
57	Kostkova et al 2013	2006-2010	UK	Infectious diseases, Clostridium difficile, MRSA, Tuberculosis, Meningitis, Norovirus, Influenza	✔						English
58	Lawson McLean et al 2016	2004-2014	Worldwide, Germany	Neurosurgery	✔						English
59	Leffler et al 2010	2004-2008	USA, UK, Canada, Australia	Ophthalmology	✔	✔			✔		English
60	Ling & Lee, 2016	2004-2015	Canada	Health Campaigns, HIV, AIDS, Stroke, Colorectal Cancer, Marijuana use	✔		✔				English
61	Linkov et al 2014	2004-2012	Worldwide, USA	Bariatric Surgery	✔				✔		English
62	Liu et al 2016	2004-2016	USA, Australia	Ankle Swelling	✔	✔			✔		English
63	Luckett et al 2016	2015	Worldwide	Chronic Breathlessness							English
64	Majumder et al 2016	2015-2016	Colombia	Zika Virus	✔				✔		Spanish
65	Mattin et al 2014	2007-2013	France, Greece, Italy, Portugal, Spain	Canine Leishmaniosis	✔						French, Greek, Italian, Portuguese, Spanish
66	Mavragani et al 2016	2004-2014	UK, Worldwide	Drugs, Prescriptions, Diclofenac, Estradiol, Macrolide Antibiotics	✔		✔				English
67	Murray et al 2016	2010-2013	Ireland	Mouth Cancer, Oral Cancer	✔					✔	English
68	Myers et al 2016	2004-2015	USA	Psychogenic Non-Epileptic Seizures	✔						English
69	Noar et al 2013	2006-2011	USA	Pancreatic Cancer, Public Figure	✔				✔	✔	English
70	Nuti et al 2014	2004-2014	Worldwide	Review	-	-	-	-	-	-	-

	Authors	Period	Region	Keywords	V	S	C	F	M	St	Languages
71	Pandey et al 2014	2004-2013	USA	Heart Transplant, Ventricular Assist Devices, Breast Cancer, Pulmonary Embolism, Bipolar Disorder, Sjogren Syndrome, Multiple Sclerosis	✔						English
72	Parker et al 2016	2010-2014	USA	Premature Deaths, Alcohol, Drugs, Suicide	✔			✔	✔		English
73	Phelan et al 2014	2009-2012	USA, UK, Australia, Ireland	Metal-on-Metal Hip	✔				✔	✔	English
74	Phelan et al 2016	2010-2015	USA	Anatomy, Education	✔	✔	✔		✔		English
75	Plante & Ingram, 2014	2004-2013	USA, Australia, Germany, UK, Canada, Sweden, Switzerland	Tinnitus Symptomatology	✔	✔					English, German, Swedish, French, Italian
76	Poletto et al 2016	2013-2015	Worldwide	Middle East Respiratory Syndrome (MERS)	✔		✔				English
77	Pollett et al 2015	2009-2014	USA	Pertussis	✔		✔	✔	✔		English
78	Rohart et al 2016	2009-2013	Australia	Disease Surveillance	✔		✔	✔	✔	✔	English
79	Rosenkrantz & Prabhu, 2016	2004-2014	USA	Imaging-Based Cancer Screening, Breast Cancer, Lung Cancer, Colon Cancer, Prostate Cancer	✔						English
80	Rossignol et al 2013	2004-2012	France, Germany, Italy, USA, China, Australia, Brazil, South Africa	Urinary Tract Infection, Cystitis	✔	✔					English, French, German, Italian, Chinese, Portuguese
81	Scatà et al 2016	2015-2016	56 countries in South America, Europe, Oceania	Epidemics, Zika Virus	✔				✔		N/A. The term is the same
82	Scheres et al 2016	2009-2015	Netherlands, Worldwide	Thrombosis, Venous Thrombosis	✔						English, Dutch
83	Shin et al 2016	2015-2016	Korea	MERS	✔		✔				Korean
84	Schootman et al 2015	2004-2014	50 US States and DC, Puerto Rico, US Virgin Islands, Guam, American Samoa, Palau	Cancer Screening	✔		✔				English

	Authors	Period	Region	Keywords	V	S	C	F	M	St	Languages
85	Schuster et al 2010	2004-2009	USA	Statins, Lipitor, Simvastatin	✔		✔		✔		English
86	Seifter et al 2010	2004-2009	USA	Lyme Disease	✔	✔					English
87	Sentana-Lledo et al 2016	2004-2014	USA	Bed bugs	✔	✔	✔				English
88	Simmering et al 2014	2004-2014	USA	Drugs, Prescriptions, Antibiotics	✔		✔				English
89	Skeldon et al 2015	2004-2007	USA	Drugs, Prostatic Hyperplasia, Avodart, Flomax	✔						English
90	Solano et al 2016	2008-2012	Italy	Suicide	✔		✔	✔			Italian
91	Stein et al 2013	2007-2010	USA, UK, Canada, India	Laser Eye Surgery	✔				✔		English
92	Takada, 2012	2004-2011	Japan	Fireflies, Beetles	✔	✔					Japanese
93	Telfer & Woodburn, 2015	2004-2014	UK, USA, Canada, Australia	Foot pain, Ankle pain, Heel pain	✔	✔			✔	✔	English
94	Troelstra et al 2016	2004-2013	Netherlands, Belgium	Tobacco Control	✔				✔	✔	Dutch
95	Toosi & Kalia, 2015	2004-2013	Canada, USA, Australia	Tanning	✔	✔					English
96	Wang et al 2015	2009-2011	Taiwan	Dementia, Alzheimer's Disease, Neurology	✔		✔	✔			Chinese
97	Warren & Wen, 2016	2004-2015	USA	Measles, MMR, Vaccine	✔						English
98	Willson et al 2015	2011-2012	USA	Aeroallergens, Allergies, Pollen	✔	✔	✔		✔		English
99	Willson et al 2015	2011-2014	USA	Pollen, Mountain Cedar	✔				✔		English
100	Yang et al 2015	2009-2015	USA	Influenza, Epidemic	✔				✔		English
101	Zhang et al 2015	2004-2014	USA, Canada, UK, Australia, China	Tobacco, Lung Cancer	✔	✔	✔			✔	English, Chinese
102	Zhang et al 2016	2004-2015	USA	Drugs, Dabbing, Cannabis Smoking	✔		✔	✔		✔	English
103	Zheluk et al 2014	2009-2013	Russia	Drugs, Krokodil, Desomorphine, Codeine	✔						Russian
104	Zhou et al 2011	2004-2009	USA	Tuberculosis	✔			✔	✔		English

References

1. Alicino C, Bragazzi NL, Faccio V, Amicizia D, Panatto D, Gasparini R, et al. Assessing Ebola-related web search behaviour: insights and implications from an analytical study of Google Trends-based query volumes. Infect Dis Poverty 2015 Dec 10;4:54 [FREE Full text] [doi: 10.1186/s40249-015-0090-9] [Medline: 26654247]
2. Arora VS, Stuckler D, McKee M. Tracking search engine queries for suicide in the United Kingdom, 2004-2013. Public Health 2016 Aug;137:147-153. [doi: 10.1016/j.puhe.2015.10.015] [Medline: 26976489]
3. Bakker KM, Martinez-Bakker ME, Helm B, Stevenson TJ. Digital epidemiology reveals global childhood disease seasonality and the effects of immunization. PNAS 2016;113(24):6689.
4. Barnes CM, Gunia BC, Wagner DT. Sleep and moral awareness. J Sleep Res 2015 Apr;24(2):181-188 [FREE Full text] [doi: 10.1111/jsr.12231] [Medline: 25159702]
5. Bentley RA, Ormerod P. Social versus independent interest in 'bird flu' and 'swine flu'. PLoS Curr 2009 Sep 3;1:RRN1036. [doi: 10.1371/currents.RRN1036]
6. Borron SW, Watts SH, Tull J, Baeza S, Diebold S, Barrow A. Intentional Misuse and Abuse of Loperamide: A New Look at a Drug with "Low Abuse Potential". J Emerg Med 2017 Jul;53(1):73-84. [doi: 10.1016/j.jemermed.2017.03.018] [Medline: 28501383]
7. Bragazzi NL. Infodemiology and infoveillance of multiple sclerosis in Italy. Mult Scler Int 2013;2013:924029 [FREE Full text] [doi: 10.1155/2013/924029] [Medline: 24027636]
8. Bragazzi NL, Dini G, Toletone A, Brigo F, Durando P. Infodemiological data concerning silicosis in the USA in the period 2004-2010 correlating with real-world statistical data. Data Brief 2017 Feb;10:457-464 [FREE Full text] [doi: 10.1016/j.dib.2016.11.021] [Medline: 28054008]
9. Bragazzi NL, Barberis I, Rosselli R, Gianfredi V, Nucci D, Moretti M, et al. How often people google for vaccination: Qualitative and quantitative insights from a systematic search of the web-based activities using Google Trends. Hum Vaccin Immunother 2017 Feb;13(2):464-469. [doi: 10.1080/21645515.2017.1264742] [Medline: 27983896]
10. Bragazzi N, Bacigaluppi S, Robba C, Siri A, Canepa G, Brigo F. Infodemiological data of West-Nile virus disease in Italy in the study period 2004-2015. Data Brief 2016:839-845 [FREE Full text]
11. Bragazzi NL, Bacigaluppi S, Robba C, Nardone R, Trinka E, Brigo F. Infodemiology of status epilepticus: A systematic validation of the Google Trends-based search queries. Epilepsy Behav 2016 Feb;55:120-123. [doi: 10.1016/j.yebeh.2015.12.017] [Medline: 26773681]
12. Bragazzi NL, Dini G, Toletone A, Brigo F, Durando P. Leveraging Big Data for Exploring Occupational Diseases-Related Interest at the Level of Scientific Community, Media Coverage and Novel Data Streams: The Example of Silicosis as a Pilot Study. PLoS One 2016;11(11):e0166051 [FREE Full text] [doi: 10.1371/journal.pone.0166051] [Medline: 27806115]
13. Bragazzi NL, Watad A, Brigo F, Adawi M, Amital H, Shoenfeld Y. Public health awareness of autoimmune diseases after the death of a celebrity. Clin Rheumatol 2016 Dec 20:1911-1917. [doi: 10.1007/s10067-016-3513-5] [Medline: 28000011]
14. Bragazzi NL. A Google Trends-based approach for monitoring NSSI. Psychol Res Behav Manag 2013 Dec;7:1-8 [FREE Full text] [doi: 10.2147/PRBM.S44084] [Medline: 24376364]
15. Braun T, Harréus U. Medical nowcasting using Google Trends: application in otolaryngology. Eur Arch Otorhinolaryngol 2013 Jul;270(7):2157-2160. [doi: 10.1007/s00405-013-2532-y] [Medline: 23632877]
16. Brigo F, Trinka E. Google search behavior for status epilepticus. Epilepsy Behav 2015 Aug;49:146-149. [doi: 10.1016/j.yebeh.2015.02.029] [Medline: 25873438]
17. Brigo F, Igwe SC, Ausserer H, Nardone R, Tezzon F, Bongiovanni LG, et al. Why do people Google epilepsy? An infodemiological study of online behavior for epilepsy-related search terms. Epilepsy Behav 2014 Feb;31:67-70. [doi: 10.1016/j.yebeh.2013.11.020] [Medline: 24361764]
18. Brigo F, Lochner P, Tezzon F, Nardone R. Web search behavior for multiple sclerosis: An infodemiological study. Multiple Sclerosis and Related Disorders 2014 Jul;3(4):440-443. [doi: 10.1016/j.msard.2014.02.005]

19. van CJS, van DE, Otte WM, Joels M, Jansen FE, Braun KPJ. Does Saint Nicholas provoke seizures? Hints from Google Trends. Epilepsy Behav 2014 Mar;32:132-134. [doi: 10.1016/j.yebeh.2014.01.019] [Medline: 24548849]
20. Carneiro HA, Mylonakis E. Google trends: a web-based tool for real-time surveillance of disease outbreaks. Clin Infect Dis 2009 Nov 15;49(10):1557-1564 [FREE Full text] [doi: 10.1086/630200] [Medline: 19845471]
21. Cavazos-Rehg PA, Krauss MJ, Spitznagel EL, Lowery A, Grucza RA, Chaloupka FJ, et al. Monitoring of non-cigarette tobacco use using Google Trends. Tob Control 2015 May;24(3):249-255 [FREE Full text] [doi: 10.1136/tobaccocontrol-2013-051276] [Medline: 24500269]
22. Cha Y, Stow CA. Mining web-based data to assess public response to environmental events. Environ Pollut 2015 Mar;198:97-99. [doi: 10.1016/j.envpol.2014.12.027] [Medline: 25577650]
23. Chaves JN, Libardi AL, Agostinho-Pesse RS, Morettin M, Alvarenga KDF. Tele-health: assessment of websites on newborn hearing screening in Portuguese Language. Codas 2015 Dec;27(6):526-533 [FREE Full text] [doi: 10.1590/2317-1782/20152014169] [Medline: 26691616]
24. Cho S, Sohn CH, Jo MW, Shin S, Lee JH, Ryoo SM, et al. Correlation between national influenza surveillance data and google trends in South Korea. PLoS One 2013 Dec;8(12):e81422 [FREE Full text] [doi: 10.1371/journal.pone.0081422] [Medline: 24339927]
25. Crowson MG, Schulz K, Tucci DL. National Utilization and Forecasting of Ototopical Antibiotics. Otology & Neurotology 2016;37(8):1049-1054. [doi: 10.1097/MAO.0000000000001115]
26. Davis NF, Gnanappiragasam S, Thornhill JA. Interstitial cystitis/painful bladder syndrome: the influence of modern diagnostic criteria on epidemiology and on Internet search activity by the public. Transl Androl Urol 2015 Oct;4(5):506-511 [FREE Full text] [doi: 10.3978/j.issn.2223-4683.2015.06.08] [Medline: 26816850]
27. Fazeli DS, Carlos RC, Hall KS, Dalton VK. Novel data sources for women's health research: mapping breast screening online information seeking through Google trends. Acad Radiol 2014 Sep;21(9):1172-1176 [FREE Full text] [doi: 10.1016/j.acra.2014.05.005] [Medline: 24998689]
28. Deiner MS, Lietman TM, McLeod SD, Chodosh J, Porco TC. Surveillance Tools Emerging From Search Engines and Social Media Data for Determining Eye Disease Patterns. JAMA Ophthalmol 2016 Sep 01;134(9):1024-1030 [FREE Full text] [doi: 10.1001/jamaophthalmol.2016.2267] [Medline: 27416554]
29. DeVilbiss E, Lee B. Brief Report: Trends in U.S. National Autism Awareness from 2004 to 2014: The Impact of National Autism Awareness Month. Journal of Autism and Developmental Disorders 2014;44(12):3271-3273. [doi: 10.1007/s10803-014-2160-4] [Medline: 24915931]
30. Domnich A, Panatto D, Signori A, Lai PL, Gasparini R, Amicizia D. Age-related differences in the accuracy of web query-based predictions of influenza-like illness. PLoS One 2015;10(5):e0127754 [FREE Full text] [doi: 10.1371/journal.pone.0127754] [Medline: 26011418]
31. El-Sheikha J. Global search demand for varicose vein information on the internet. Phlebology 2015 Sep;30(8):533-540. [doi: 10.1177/0268355514542681] [Medline: 24993972]
32. Fenichel EP, Kuminoff NV, Chowell G. Skip the trip: air travelers' behavioral responses to pandemic influenza. PLoS One 2013 Mar;8(3):e58249 [FREE Full text] [doi: 10.1371/journal.pone.0058249] [Medline: 23526970]
33. Fond G, Gaman A, Brunel L, Haffen E, Llorca P. Google Trends ® : Ready for real-time suicide prevention or just a Zeta-Jones effect? An exploratory study. Psychiatry Research 2015 Aug;228(3):913-917. [doi: 10.1016/j.psychres.2015.04.022]
34. Foroughi F, Lam AK, Lim MS, Saremi N, Ahmadvand A. "Googling" for Cancer: An Infodemiological Assessment of Online Search Interests in Australia, Canada, New Zealand, the United Kingdom, and the United States. JMIR Cancer 2016 May 04;2(1):e5 [FREE Full text] [doi: 10.2196/cancer.5212] [Medline: 28410185]
35. Gafson AR, Giovannoni G. CCSVI-A. A call to clinicans and scientists to vocalise in an Internet age. Mult Scler Relat Disord 2014 Mar;3(2):143-146. [doi: 10.1016/j.msard.2013.10.005] [Medline: 25878001]

36. Gahr M, Uzelac Z, Zeiss R, Connemann BJ, Lang D, Schönfeldt-Lecuona C. Linking Annual Prescription Volume of Antidepressants to Corresponding Web Search Query Data: A Possible Proxy for Medical Prescription Behavior? J Clin Psychopharmacol 2015 Dec;35(6):681-685. [doi: 10.1097/JCP.0000000000000397] [Medline: 26355849]
37. Gamma A, Schleifer R, Weinmann W, Buadze A, Liebrenz M. Could Google Trends Be Used to Predict Methamphetamine-Related Crime? An Analysis of Search Volume Data in Switzerland, Germany, and Austria. PLoS ONE 2016 Nov 30;11(11):e0166566. [doi: 10.1371/journal.pone.0166566]
38. Garrison SR, Dormuth CR, Morrow RL, Carney GA, Khan KM. Seasonal effects on the occurrence of nocturnal leg cramps: a prospective cohort study. CMAJ 2015 Mar 03;187(4):248-253 [FREE Full text] [doi: 10.1503/cmaj.140497] [Medline: 25623650]
39. Gollust SE, Qin X, Wilcock AD, Baum LM, Barry CL, Niederdeppe J, et al. Search and You Shall Find: Geographic Characteristics Associated With Google Searches During the Affordable Care Act's First Enrollment Period. Med Care Res Rev 2017 Dec;74(6):723-735. [doi: 10.1177/1077558716660944] [Medline: 27457426]
40. Guernier V, Milinovich GJ, Bezerra SMA, Haworth M, Coleman G, Soares MRJ. Use of big data in the surveillance of veterinary diseases: early detection of tick paralysis in companion animals. Parasit Vectors 2016 Dec 23;9(1):303 [FREE Full text] [doi: 10.1186/s13071-016-1590-6] [Medline: 27215214]
41. Haney NM, Kinsella SD, Morey JM. United States medical school graduate interest in radiology residency programs as depicted by online search tools. J Am Coll Radiol 2014 Feb;11(2):193-197. [doi: 10.1016/j.jacr.2013.06.023] [Medline: 24120904]
42. Harorli OT, Harorli H. Evaluation of internet search trends of some common oral problems, 2004 to 2014. Community Dental Health 2014;31(3):188-192. [doi: 10.1922/CDH_3330Harorl?05]
43. Harsha AK, Schmitt JE, Stavropoulos SW. Know your market: use of online query tools to quantify trends in patient information-seeking behavior for varicose vein treatment. J Vasc Interv Radiol 2014 Jan;25(1):53-57. [doi: 10.1016/j.jvir.2013.09.015] [Medline: 24286941]
44. Harsha AK, Schmitt JE, Stavropoulos SW. Match day: online search trends reflect growing interest in IR training. J Vasc Interv Radiol 2015 Jan;26(1):95-100. [doi: 10.1016/j.jvir.2014.09.011] [Medline: 25541447]
45. Hassid BG, Day LW, Awad MA, Sewell JL, Osterberg EC, Breyer BN. Using Search Engine Query Data to Explore the Epidemiology of Common Gastrointestinal Symptoms. Dig Dis Sci 2017 Dec;62(3):588-592. [doi: 10.1007/s10620-016-4384-y] [Medline: 27878646]
46. Hossain L, Kam D, Kong F, Wigand RT, Bossomaier T. Social media in Ebola outbreak. Epidemiol Infect 2016 Jul;144(10):2136-2143. [doi: 10.1017/S095026881600039X] [Medline: 26939535]
47. Huang J, Zheng R, Emery S. Assessing the impact of the national smoking ban in indoor public places in china: evidence from quit smoking related online searches. PLoS One 2013 Jun;8(6):e65577 [FREE Full text] [doi: 10.1371/journal.pone.0065577] [Medline: 23776504]
48. Huesch M, Chetlen A, Segel J, Schetter S. Frequencies of Private Mentions and Sharing of Mammography and Breast Cancer Terms on Facebook: A Pilot Study. J Med Internet Res 2017 Jun 09;19(6):e201 [FREE Full text] [doi: 10.2196/jmir.7508] [Medline: 28600279]
49. Ingram DG, Plante DT. Seasonal trends in restless legs symptomatology: evidence from Internet search query data. Sleep Med 2013 Dec;14(12):1364-1368. [doi: 10.1016/j.sleep.2013.06.016] [Medline: 24152798]
50. Ingram DG, Matthews CK, Plante DT. Seasonal trends in sleep-disordered breathing: evidence from Internet search engine query data. Sleep Breath 2015 Mar;19(1):79-84. [doi: 10.1007/s11325-014-0965-1] [Medline: 24595717]
51. Jha S, Wang Z, Laucis N, Bhattacharyya T. Trends in Media Reports, Oral Bisphosphonate Prescriptions, and Hip Fractures 1996-2012: An Ecological Analysis. J Bone Miner Res 2015 Dec;30(12):2179-2187 [FREE Full text] [doi: 10.1002/jbmr.2565] [Medline: 26018247]

52. Johnson AK, Mehta SD. A comparison of Internet search trends and sexually transmitted infection rates using Google trends. Sex Transm Dis 2014 Jan;41(1):61-63. [doi: 10.1097/OLQ.0000000000000065] [Medline: 24326584]
53. Kadry B, Chu LF, Kadry B, Gammas D, Macario A. Analysis of 4999 online physician ratings indicates that most patients give physicians a favorable rating. J Med Internet Res 2011 Nov;13(4):e95 [FREE Full text] [doi: 10.2196/jmir.1960] [Medline: 22088924]
54. Kang M, Zhong H, He J, Rutherford S, Yang F. Using Google Trends for influenza surveillance in South China. PLoS One 2013;8(1):e55205 [FREE Full text] [doi: 10.1371/journal.pone.0055205] [Medline: 23372837]
55. Kang M, Song W, Choi S, Kim H, Ha H, Kim S, et al. Google unveils a glimpse of allergic rhinitis in the real world. Allergy 2015 Jan;70(1):124-128. [doi: 10.1111/all.12528] [Medline: 25280183]
56. Koburger N, Mergl R, Rummel-Kluge C, Ibelshäuser A, Meise U, Postuvan V, et al. Celebrity suicide on the railway network: Can one case trigger international effects? J Affect Disord 2015 Oct 01;185:38-46. [doi: 10.1016/j.jad.2015.06.037] [Medline: 26143403]
57. Kostkova P, Fowler D, Wiseman S, Weinberg JR. Major infection events over 5 years: how is media coverage influencing online information needs of health care professionals and the public? J Med Internet Res 2013 Jul 15;15(7):e107 [FREE Full text] [doi: 10.2196/jmir.2146] [Medline: 23856364]
58. Lawson MAC, Lawson MA, Kalff R, Walter J. Google Search Queries About Neurosurgical Topics: Are They a Suitable Guide for Neurosurgeons? World Neurosurg 2016 Jun;90:179-185. [doi: 10.1016/j.wneu.2016.02.045] [Medline: 26898496]
59. Leffler CT, Davenport B, Chan D. Frequency and seasonal variation of ophthalmology-related internet searches. Can J Ophthalmol 2010 Jun;45(3):274-279. [doi: 10.3129/i10-022] [Medline: 20436544]
60. Ling R, Lee J. Disease Monitoring and Health Campaign Evaluation Using Google Search Activities for HIV and AIDS, Stroke, Colorectal Cancer, and Marijuana Use in Canada: A Retrospective Observational Study. JMIR Public Health Surveill 2016 Oct 12;2(2):e156 [FREE Full text] [doi: 10.2196/publichealth.6504] [Medline: 27733330]
61. Linkov F, Bovbjerg DH, Freese KE, Ramanathan R, Eid GM, Gourash W. Bariatric surgery interest around the world: what Google Trends can teach us. Surg Obes Relat Dis 2014 May;10(3):533-538. [doi: 10.1016/j.soard.2013.10.007] [Medline: 24794184]
62. Liu F, Allan GM, Korownyk C, Kolber M, Flook N, Sternberg H, et al. Seasonality of Ankle Swelling: Population Symptom Reporting Using Google Trends. Ann Fam Med 2016 Dec;14(4):356-358 [FREE Full text] [doi: 10.1370/afm.1953] [Medline: 27401424]
63. Luckett T, Disler R, Hosie A, Johnson M, Davidson P, Currow D, et al. Content and quality of websites supporting self-management of chronic breathlessness in advanced illness: a systematic review. NPJ Prim Care Respir Med 2016 Dec 26;26:16025 [FREE Full text] [doi: 10.1038/npjpcrm.2016.25] [Medline: 27225898]
64. Majumder MS, Santillana M, Mekaru SR, McGinnis DP, Khan K, Brownstein JS. Utilizing Nontraditional Data Sources for Near Real-Time Estimation of Transmission Dynamics During the 2015-2016 Colombian Zika Virus Disease Outbreak. JMIR Public Health Surveill 2016 Jun 01;2(1):e30 [FREE Full text] [doi: 10.2196/publichealth.5814] [Medline: 27251981]
65. Mattin MJ, Solano-Gallego L, Dhollander S, Afonso A, Brodbelt DC. The frequency and distribution of canine leishmaniosis diagnosed by veterinary practitioners in Europe. Vet J 2014 Jun;200(3):410-419. [doi: 10.1016/j.tvjl.2014.03.033] [Medline: 24767097]
66. Mavragani A, Sypsa K, Sampri A, Tsagarakis K. Quantifying the UK Online Interest in Substances of the EU Watchlist for Water Monitoring: Diclofenac, Estradiol, and the Macrolide Antibiotics. Water 2016 Nov 18;8(11):542. [doi: 10.3390/w8110542]
67. Murray G, O'Rourke C, Hogan J, Fenton JE. Detecting internet search activity for mouth cancer in Ireland. Br J Oral Maxillofac Surg 2016 Feb;54(2):163-165. [doi: 10.1016/j.bjoms.2015.12.005] [Medline: 26774361]

68. Myers L, Jones J, Boesten N, Lancman M. Psychogenic non-epileptic seizures (PNES) on the Internet: Online representation of the disorder and frequency of search terms. Seizure 2016 Aug 01;40:114-122 [FREE Full text]
69. Noar S, Ribisl K, Althouse B, Willoughby J, Ayers J. Using digital surveillance to examine the impact of public figure pancreatic cancer announcements on media and search query outcomes. Journal of the National Cancer Institute - Monographs 2013:188-194.
70. Nuti SV, Wayda B, Ranasinghe I, Wang S, Dreyer RP, Chen SI, et al. The use of google trends in health care research: a systematic review. PLoS One 2014 Oct;9(10):e109583 [FREE Full text] [doi: 10.1371/journal.pone.0109583] [Medline: 25337815]
71. Pandey A, Abdullah K, Drazner MH. Impact of Vice President Cheney on public interest in left ventricular assist devices and heart transplantation. Am J Cardiol 2014 May 01;113(9):1529-1531. [doi: 10.1016/j.amjcard.2014.02.007] [Medline: 24630787]
72. Parker J, Cuthbertson C, Loveridge S, Skidmore M, Dyar W. Forecasting state-level premature deaths from alcohol, drugs, and suicides using Google Trends data. J Affect Disord 2017 Dec 15;213:9-15. [doi: 10.1016/j.jad.2016.10.038] [Medline: 28171770]
73. Phelan N, Kelly JC, Moore DP, Kenny P. The effect of the metal-on-metal hip controversy on Internet search activity. Eur J Orthop Surg Traumatol 2014 Oct;24(7):1203-1210. [doi: 10.1007/s00590-013-1399-3] [Medline: 24390041]
74. Phelan N, Davy S, O'Keeffe GW, Barry DS. Googling in anatomy education: Can google trends inform educators of national online search patterns of anatomical syllabi? Anat Sci Educ 2017 Mar;10(2):152-159. [doi: 10.1002/ase.1641] [Medline: 27547967]
75. Plante DT, Ingram DG. Seasonal trends in tinnitus symptomatology: evidence from Internet search engine query data. Eur Arch Otorhinolaryngol 2015 Oct;272(10):2807-2813. [doi: 10.1007/s00405-014-3287-9] [Medline: 25234771]
76. Poletto C, Boëlle P, Colizza V. Risk of MERS importation and onward transmission: a systematic review and analysis of cases reported to WHO. BMC Infect Dis 2016 Aug 25;16(1):448 [FREE Full text] [doi: 10.1186/s12879-016-1787-5] [Medline: 27562369]
77. Pollett S, Wood N, Boscardin WJ, Bengtsson H, Schwarcz S, Harriman K, et al. Validating the Use of Google Trends to Enhance Pertussis Surveillance in California. PLoS Curr 2015 Oct 19:1-10. [doi: 10.1371/currents.outbreaks.7119696b3e7523faa4543faac87c56c2]
78. Rohart F, Milinovich GJ, Avril SMR, Lê CK, Tong S, Hu W. Disease surveillance based on Internet-based linear models: an Australian case study of previously unmodeled infection diseases. Sci Rep 2016 Dec 20;6:38522 [FREE Full text] [doi: 10.1038/srep38522] [Medline: 27994231]
79. Rosenkrantz AB, Prabhu V. Public Interest in Imaging-Based Cancer Screening Examinations in the United States: Analysis Using a Web-Based Search Tool. AJR Am J Roentgenol 2016 Jan;206(1):113-118. [doi: 10.2214/AJR.15.14840] [Medline: 26700342]
80. Rossignol L, Pelat C, Lambert B, Flahault A, Chartier-Kastler E, Hanslik T. A method to assess seasonality of urinary tract infections based on medication sales and google trends. PLoS One 2013;8(10):e76020 [FREE Full text] [doi: 10.1371/journal.pone.0076020] [Medline: 24204587]
81. Scatà M, Di SA, Liò P, La CA. The Impact of Heterogeneity and Awareness in Modeling Epidemic Spreading on Multiplex Networks. Sci Rep 2016 Dec 16;6:37105 [FREE Full text] [doi: 10.1038/srep37105] [Medline: 27848978]
82. Scheres LJJ, Lijfering WM, Middeldorp S, Cannegieter SC. Influence of World Thrombosis Day on digital information seeking on venous thrombosis: a Google Trends study. J Thromb Haemost 2016 Dec;14(12):2325-2328. [doi: 10.1111/jth.13529] [Medline: 27735128]
83. Shin S, Seo D, An J, Kwak H, Kim S, Gwack J, et al. High correlation of Middle East respiratory syndrome spread with Google search and Twitter trends in Korea. Sci Rep 2016 Sep 06;6:32920 [FREE Full text] [doi: 10.1038/srep32920] [Medline: 27595921]
84. Schootman M, Toor A, Cavazos-Rehg P, Jeffe DB, McQueen A, Eberth J, et al. The utility of Google Trends data to examine interest in cancer screening. BMJ Open 2015 Jun 08;5(6):e006678 [FREE Full text] [doi: 10.1136/bmjopen-2014-006678] [Medline: 26056120]

85. Schuster N, Rogers M, McMahon JL. Using search engine query data to track pharmaceutical utilization: a study of statins. The American journal of managed care 2010;16(8):215-219.
86. Seifter A, Schwarzwalder A, Geis K, Aucott J. The utility of "Google Trends" for epidemiological research: Lyme disease as an example. Geospat Health 2010 May;4(2):135-137. [doi: 10.4081/gh.2010.195] [Medline: 20503183]
87. Sentana-Lledo D, Barbu CM, Ngo MN, Wu Y, Sethuraman K, Levy MZ. Seasons, Searches, and Intentions: What The Internet Can Tell Us About The Bed Bug (Hemiptera: Cimicidae) Epidemic. J Med Entomol 2016 Jan;53(1):116-121. [doi: 10.1093/jme/tjv158] [Medline: 26474879]
88. Simmering JE, Polgreen LA, Polgreen PM. Web search query volume as a measure of pharmaceutical utilization and changes in prescribing patterns. Res Social Adm Pharm 2014;10(6):896-903. [doi: 10.1016/j.sapharm.2014.01.003] [Medline: 24603135]
89. Skeldon SC, Kozhimannil KB, Majumdar SR, Law MR. The effect of competing direct-to-consumer advertising campaigns on the use of drugs for benign prostatic hyperplasia: time series analysis. J Gen Intern Med 2015 Apr;30(4):514-520 [FREE Full text] [doi: 10.1007/s11606-014-3063-y] [Medline: 25338730]
90. Solano P, Ustulin M, Pizzorno E, Vichi M, Pompili M, Serafini G, et al. A Google-based approach for monitoring suicide risk. Psychiatry Res 2016 Dec 30;246:581-586. [doi: 10.1016/j.psychres.2016.10.030] [Medline: 27837725]
91. Stein JD, Childers DM, Nan B, Mian SI. Gauging interest of the general public in laser-assisted in situ keratomileusis eye surgery. Cornea 2013 Jul;32(7):1015-1018 [FREE Full text] [doi: 10.1097/ICO.0b013e318283c85a] [Medline: 23538615]
92. Takada K. Japanese Interest in "Hotaru" (Fireflies) and "Kabuto-Mushi" (Japanese Rhinoceros Beetles) Corresponds with Seasonality in Visible Abundance. Insects 2012 Apr 10;3(2):424-431 [FREE Full text] [doi: 10.3390/insects3020424] [Medline: 26466535]
93. Telfer S, Woodburn J. Let me Google that for you: a time series analysis of seasonality in internet search trends for terms related to foot and ankle pain. J Foot Ankle Res 2015 Jul;8:27 [FREE Full text] [doi: 10.1186/s13047-015-0074-9] [Medline: 26146521]
94. Troelstra SA, Bosdriesz JR, de BMR, Kunst AE. Effect of Tobacco Control Policies on Information Seeking for Smoking Cessation in the Netherlands: A Google Trends Study. PLoS One 2016 Feb;11(2):e0148489 [FREE Full text] [doi: 10.1371/journal.pone.0148489] [Medline: 26849567]
95. Toosi B, Kalia S. Seasonal and Geographic Patterns in Tanning Using Real-Time Data From Google Trends. JAMA Dermatol 2016 Feb;152(2):215-217. [doi: 10.1001/jamadermatol.2015.3008] [Medline: 26719968]
96. Wang H, Chen D, Yu H, Chen Y. Forecasting the Incidence of Dementia and Dementia-Related Outpatient Visits With Google Trends: Evidence From Taiwan. J Med Internet Res 2015 Nov 19;17(11):e264 [FREE Full text] [doi: 10.2196/jmir.4516] [Medline: 26586281]
97. Warren KE, Wen LS. Measles, social media and surveillance in Baltimore City. J Public Health (Oxf) 2017 Sep 01;39(3):e73-e78. [doi: 10.1093/pubmed/fdw076] [Medline: 27521926]
98. Willson TJ, Lospinoso J, Weitzel E, McMains K. Correlating Regional Aeroallergen Effects on Internet Search Activity. Otolaryngol Head Neck Surg 2014 Dec 12;152(2):228-232. [doi: 10.1177/0194599814560149] [Medline: 25505261]
99. Willson TJ, Shams A, Lospinoso J, Weitzel E, McMains K. Searching for Cedar: Geographic Variation in Single Aeroallergen Shows Dose Response in Internet Search Activity. Otolaryngol Head Neck Surg 2015 Nov 02;153(5):770-774. [doi: 10.1177/0194599815601650] [Medline: 26340925]
100. Yang S, Santillana M, Kou SC. Accurate estimation of influenza epidemics using Google search data via ARGO. PNAS 2015;112(47):14473.
101. Zhang Z, Zheng X, Zeng DD, Leischow SJ. Information seeking regarding tobacco and lung cancer: effects of seasonality. PLoS One 2015 Mar;10(3):e0117938 [FREE Full text] [doi: 10.1371/journal.pone.0117938] [Medline: 25781020]

102. Zhang Z, Zheng X, Zeng DD, Leischow SJ. Tracking Dabbing Using Search Query Surveillance: A Case Study in the United States. J Med Internet Res 2016 Sep 16;18(9):e252 [FREE Full text] [doi: 10.2196/jmir.5802] [Medline: 27637361]
103. Zheluk A, Quinn C, Meylakhs P. Internet search and krokodil in the Russian Federation: an infoveillance study. J Med Internet Res 2014 Sep 18;16(9):e212 [FREE Full text] [doi: 10.2196/jmir.3203] [Medline: 25236385]
104. Zhou X, Ye J, Feng Y. Tuberculosis surveillance by analyzing Google trends. IEEE Trans Biomed Eng 2011 Aug;58(8):2247-2254. [doi: 10.1109/TBME.2011.2132132] [Medline: 21435969]
